# What do We Know about Cryptic Aspergillosis?

**DOI:** 10.3390/microorganisms12050886

**Published:** 2024-04-28

**Authors:** Nicholas Geremia, Federico Giovagnorio, Agnese Colpani, Andrea De Vito, Giorgia Caruana, Maria Chiara Meloni, Giordano Madeddu, Sandro Panese, Saverio Giuseppe Parisi

**Affiliations:** 1 Unit of Infectious Diseases, Department of Clinical Medicine, Ospedale Dell’Angelo, 30174 Venice, Italy; sandro.panese@aulss3.veneto.it; 2 Unit of Infectious Diseases, Department of Clinical Medicine, Ospedale Civile “S.S. Giovanni e Paolo”, 30122 Venice, Italy; 3 Department of Molecular Medicine, University of Padua, 35121 Padua, Italy; federico.giovagnorio@studenti.unipd.it (F.G.); saverio.parisi@unipd.it (S.G.P.); 4 Unit of Infectious Diseases, Department of Medicine, Surgery and Pharmacy, University of Sassari, 07100 Sassari, Italy; colpaniagnese@gmail.com (A.C.); andreadevitoaho@gmail.com (A.D.V.); m_chiara@hotmail.it (M.C.M.); giordano@uniss.it (G.M.); 5 Biomedical Science Department, School in Biomedical Science, University of Sassari, 07100 Sassari, Italy; 6 Department of Laboratory Medicine and Pathology, Institute of microbiology, Lausanne University Hospital and University of Lausanne, 1011 Lausanne, Switzerland; giorgia.caruana@chuv.ch; 7 Infectious Diseases Service, Department of Medicine, Lausanne University Hospital and University of Lausanne, 1011 Lausanne, Switzerland

**Keywords:** *Aspergillus*, cryptic aspergillosis, one health, antifungal treatments, antifungal resistances

## Abstract

Cryptic *Aspergillus* species are increasingly recognized as pathogens involved in human disease. They are ubiquitarian fungi with high tenacity in their environment and can express various resistance mechanisms, often due to exposure to antifungal agents employed in agriculture and farming. The identification of such species is increasing thanks to molecular techniques, and a better description of this type of pathogen is granted. Nevertheless, the number of species and their importance in the clinical setting still need to be well studied. Furthermore, their cross-sectional involvement in animal disease, plants, and human activities requires a multidisciplinary approach involving experts from various fields. This comprehensive review aims to provide a sharp vision of the cryptic *Aspergillus* species, from the importance of correct identification to the better management of the infections caused by these pathogens. The review also accentuates the importance of the One Health approach for this kind of microorganism, given the interconnection between environmental exposure and aspergillosis, embracing transversely the multidisciplinary process for managing the cryptic *Aspergillus* species. The paper advocates the need for improving knowledge in this little-known species, given the burden of economic and health implications related to the diffusion of these bugs.

## 1. Introduction

*Aspergillus* species are filamentous molds that function as saprophytes, asymptomatic endophytes, and opportunistic phytopathogens. They play a significant role in the degradation processes of organic matter in ecosystems, and they can be found in a variety of substrata of major biofilms, including soil, food, and litter, with ubiquitous spores [1,2,3]. The *Aspergillus* species (spp.) known until now are reported to be between 300 and 400 [4]. In the family of *Aspergillaceae*, only five sections are reported to cause human disease: *Fumigati*, *Flavi*, *Nigri*, *Terrei*, and *Nidulante* [5].

Over the last decade, infections caused by filamentous fungi have increased in number and pathogenicity, resulting in major life-threatening causes, especially in immunocompromised hosts [6]. *Aspergillus* spp. is the most frequent mold isolated in clinical samples [7]. Human aspergillosis involves a wide spectrum of clinical presentations, ranging from pulmonary infections (e.g., invasive pulmonary aspergillosis) to different forms of hypersensitivity (e.g., allergic bronchopulmonary aspergillosis) [8,9]. In humans, *Aspergillus fumigatus* is the most frequent fungal pathogen, followed by *A. flavus*, *A. terreus*, and *A. niger* [3,10,11,12].

There is a complex relationship between human, environmental, and occupational exposure to *Aspergillus*. Some species are known to be able to produce mycotoxins [13,14], which can contaminate food, like crops [15], causing major public health issues with human implications, such as food contamination by aflatoxin, which occurred in Kenya, where 125 deaths were recorded in 2005 [16]. Indoor plants represent a natural reservoir for the proliferation of these fungi [17], and several studies have reported a correlation between exposure to indoor, air-borne fungi produced in buildings damaged by moisture and adverse health effects [18,19]. Finally, occupational exposure has been studied, reporting various links between jobs and potential health problems related to *Aspergillus* species’ exposition [20,21].

Thanks to technological advances, new species among the *Aspergillus* family have been identified [22,23]. Scientists define this type of fungi as a “cryptic” or “sibling” species because they are indistinguishable from each other by classical identification tools usually employed in standard laboratories. Another definition is “species complex”, a species closely related and indistinguishable by morphological methods [24]. They are grouped into a single species complex called a “section”. Beyond taxonomic classification, the clinical interest in these cryptic species is rising. It is estimated that between 10% and 30% of invasive aspergillosis (IA) is caused by cryptic species [25,26,27].

The clinical importance of cryptic species is arising. The high risk of azole-resistance or pan-antifungal resistance, especially in cryptic aspergillosis, can have a profound impact on mortality and morbidity, especially in immunocompromised patients where the risk of developing invasive aspergillosis is much higher than in the general population [28,29].

## 2. Materials and Methods

A comprehensive literature search was conducted to identify relevant studies and articles about cryptic aspergillosis. The search was not restricted by language or publication date. The keywords and MeSH terms included “Cryptic aspergillosis”, “Invasive aspergillosis”, “Taxonomy AND aspergillosis”, “Therapy strategies AND aspergillosis”, “Antifungal resistance AND Aspergillosis”, “Antifungal resistance and cryptic aspergillosis”, “Azole resistance AND cryptic aspergillosis”, “Amphotericin B resistance AND cryptic aspergillosis”, “Systematic review AND cryptic aspergillosis”, “Diagnosis AND aspergillosis”, “Environment AND aspergillosis” and “Environment AND cryptic aspergillosis”. We screened the articles by title, keywords, abstract, and full text. After an initial screening of titles and abstracts of published articles, the reviewers evaluated full articles to assess eligibility for each study’s inclusion in this narrative review. The decision to include any examined study was determined if it was likely to provide valid and valuable information according to the review’s objective.

## 3. Taxonomy

*Aspergillus* spp. is a genus that encloses many species present worldwide. The classification and identification have been based mainly on phenotypic characters. Still, in the last decades, molecular and chemotaxonomic characterization has played a crucial role in developing a complete picture of this type of fungi.

The basis of classification was laid by Raper and Fennell in 1965: they divided the genus into 18 groups [30]. Gams et al. in 1985 introduced names of subgenera and sections in *Aspergillus* [31]. Various authors contributed to widening the subgenera and sections [32]. The morphological characterization of *Aspergillus* mostly follows the protocols of Raper and Fennell, Klich, Pitt and Hocking, and Samson et al. [30,33,34,35]. The phenotype-based infrageneric classification was primarily based on conidium color, conidiophore morphology, and growth rates [36]. Some of the morphological characters chosen by many authors are the shapes and sizes of various structures of the fungi [37]. Physiological characteristics are fundamental to describing cryptic species, such as colony diam, production of colored metabolites, growth rates, and the production of extrolites [37].

Alongside the morphological and physiological description, the use of the internal transcribed spacers (ITS) of the Nuclear Ribosomal DNA (nrDNA), now accepted as the official DNA barcode for fungi [38], is primarily used mainly for phylogenetic species recognition [32].

Other essential tools are multi-locus sequencing of calmodulin and partially of β-tubulin, a DNA-based method of identification, and matrix-assisted laser desorption/ionization (MALDI-TOF MS), that allows us to correctly recognize cryptic species among the *Aspergillus* genus [3,39,40,41]. Morphological and physiological classification greatly overlaps with the current DNA-based classification in *Aspergillus* [36]. However, *Aspergillus* ITS sequences are identical in several complexes of important species; hence, more DNA markers are necessary [37].

The clades in this genus have a genetic distance larger than those seen in other fungal genera [32,42]. Using infrageneric ranks for phylogenetic clades helps manage species genera like *Aspergillus* [36]. Furthermore, the categorization between genus and species level, in addition to being official nomenclature taxonomic ranks, is also highly predictive of what functional characters the species might have [36].

**Figure 1:**
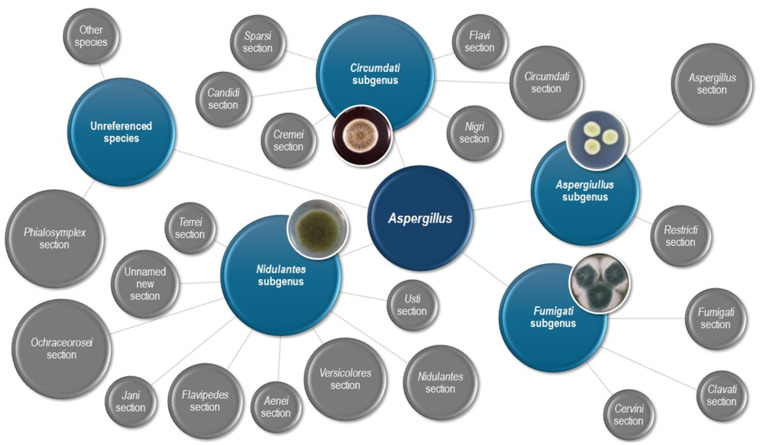
Classification according to subgenus and section of *Aspergillus* genus.

An essential change in nomenclature was made in 2012 when the concept of “one fungus, one name” was established [43,44]. This is important, since the ascomycete anamorph genus *Aspergillus* encompasses at least ten teleomorph genera more carefully delimitated than the anamorph genus [32,42]. Phylogenetic studies have shown that *Aspergillus* forms a monophyletic clade that is strictly related to *Penicillium* [3,32,42]. This finding, alongside the absence of precise criteria for the genetic delimitation of the genus in Kingdom Fungi, leads to the retention of the genus *Aspergillus* in the broadest spectrum (with all teleomorph genera assumed). The decision is supported by the Commission of *Penicillium* and *Aspergillus* [32,45,46,47]. The genus *Aspergillus* is divided by subgenus and sections [48]. Cryptic species must be considered as independent evolutionary lines [48]. In Figure 1, we report the classification of the *Aspergillus* genus.

*A. lentulus* was first described in 2005 as a sibling species of *A. fumigatus*, identified in a case of human aspergillosis [22]. In 2005, another cryptic species of the *A. fumigatus* complex, *A. pseudofischeri*, was proven to have been misidentified as *A. fumigatus* [39]. Since the first identifications, several cryptic species have also been identified in other *Aspergillus* groups, such as *A. niger* and *A. ustus* species complexes [24].

The most frequently reported cryptic species that cause infections in humans are shown in Table 1 [3,49].

For instance, of the 60 phylogenetically distinct species of *Aspergillus* section *Fumigati*, approximately 20 were reported to cause infections both in humans and animals [50,51]. *Aspergillus* section *Nidulantes* series *Versicolores* has been proposed to contain 18 species, mainly cryptic, many of which are opportunistic pathogens [52]. Similar complexity is attributable to the sections *Nigri* and *Flavi* [53,54].

However, new molecular techniques also permit unnecessary intricacies to be avoided. Previously named species were more accurately reassigned to the old ones as synonymous, reducing the number of species [55]. This was due to the better correlation with intraspecific variation previously reported for other aspergilli. Using proteomic or DNA-sequence-based identification methods allowed for reassignment and simplification [55,56].

## 4. One-Health Approach

The role of *Aspergillus* spp. as an animal pathogen and its ubiquitarian distribution and tenacity in the environment make these fungi an ideal target of One Health approaches. The awareness of its cross-sectional impact is crucial to designing target intervention and control strategy; for example, the increased report of azole resistance has been connected to using fungicides and pesticides in agriculture and farming. Knowing the underlying mechanism and cooperating with experts in the field may help reduce the clinical side effects of this practice.

### 4.1. Agriculture

*Aspergillus* spp. thrives on decaying plant material but can grow on or in plants and trees. It can be found in a wide variety of environments and throughout the whole year [57]. The wide use of pesticides, even if not targeted to *Aspergillus* spp., can quickly impact its resistance profile. The postulated mechanism is the similarity in structure and molecular target Cyp51A of triazole used in agriculture and farming and the azoles used in clinical practice [58]. The isolation of resistant strains in the environment supports the hypothesis that part of the clinically relevant resistance mechanism may be acquired from the surroundings [59]. Further epidemiological and genetic studies have brought more evidence supporting this hypothesis [60].

**Figure 2:**
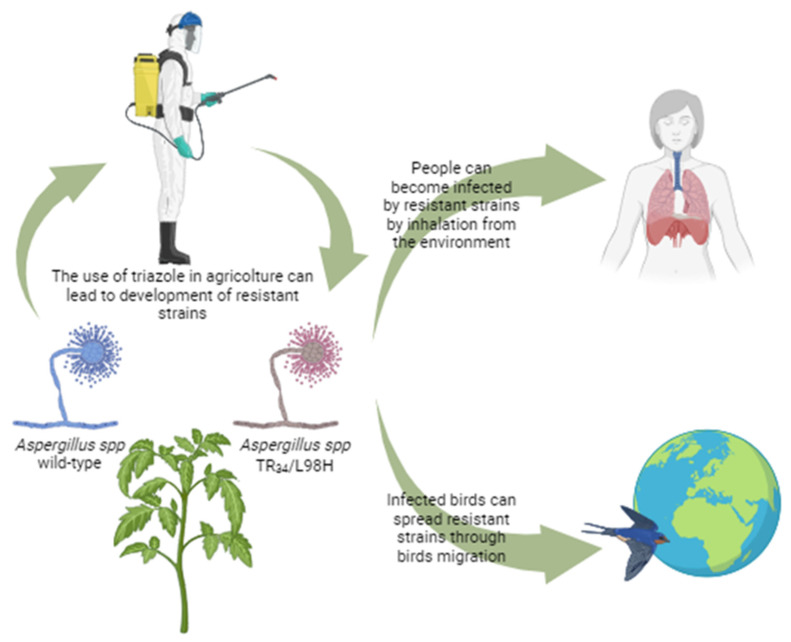
Transmission of azole-resistant aspergillosis using triazole in agriculture.

The primary mutation responsible for azole resistance highlighted so far is the TR_34_/L98H on the Cyp51A gene, which has shown similar fitness and virulence to the wild-type strain [60]. Nevertheless, once the role of pesticides and fungicides has been established, how to turn the tide is yet to be clarified. Despite being regulated in high-income countries, many nations do not have any policy regarding the use of pesticides and the permitted levels in water and soil [61,62]. The pathogenic role of *Aspergillus* spp. on plants and the threat of mycotoxins for humans is well known. Yet alternative control methods are lacking, and chemical fungicides are still the primary tool available [63]. Transmission of azole-resistant aspergillosis using triazole in agriculture is shown in Figure 2.

### 4.2. Animals

Similarly to humans, birds can become infected with *Aspergillosis* spp. by inhalation of conidia. The infection can profoundly affect birds’ health, or they can remain completely asymptomatic. They can spread the pathogen through their intestinal system and carcass. Given the high distance birds can cover during migration, their role as spreaders of *Aspergillus* spp. strains, including those carrying azole resistance mutations, have been increasingly recognized. Human migration and air movements are also possible mechanisms; however, bird migration seems to be the most likely reason for identifying identical strains in geographical areas far apart [64].

Not only birds have a role in the dispersion of resistant strains acquired from the environment, but fungicides in wild birds’ prophylaxis and poultry house disinfection may trigger azole resistance mutations in this species [60,61]. Another reason for the attention given to the avian population is their role as markers of the environment. For example, investigating the prevalence of resistant strains among birds could be a reliable proxy for the actual prevalence of these strains in the environment [65]. It is of note that *Aspergillus* spp. can thrive in various animals, including vertebrates and invertebrates, from corals to cattle. *A. viridinutans* species complex, a group of cryptic species, has been increasingly associated with animal as well as human infections [66]. However, the role of other animal species in One Health’s vision seems less critical [57].

### 4.3. Cryptic Aspergillosis and Reservoirs

Cryptic aspergillosis is a growing problem all over the world. Emerging new species of *Aspergillus* from tropical and subtropical areas confirm their evolutionary advantage in the face of climate change, thus suggesting that cryptic species will be more and more common in the future [67]. Cryptic species threaten humans and animals due to higher Minimum Inhibitory Concentrations (MICs) in antifungal agents and challenging diagnosis [68,69].

Many cryptic species have been identified as human pathogens, but they can also affect animals; for example, *A. felis* has been highlighted as the leading cause of aspergillosis in cats [70], but human case reports have also been reported [71]. *A. alabamensis* is one of the cryptic species described in dogs and humans [72,73].

Moreover, similarly to non-cryptic species, they are ubiquitously distributed and can be found in a wide range of environments; an example of the impact of these species on agricultural settings is the role of *A. novoparasiticus* as a contaminant of sugar cane production chain [74], rice and corn fields [75,76].

Despite the lack of robust data on cryptic species, case reports and limited published studies suggest a tight entanglement between medical, veterinary, and environmental sciences. Nonetheless, more epidemiological data are needed to assess the impact of these species on human, animal, and environmental Health. Thus, the One Health approach should be encouraged when conducting further research to better understand and control cryptic species.

## 5. Diagnosis and Identification

Diagnostic methods constitute the bedrock of unraveling cryptic aspergillosis.

Culture, the cornerstone of microbiological identification, unveils the fungal phenotypical characteristics. *Aspergillus*, a filamentous fungus, thrives in various cultural media. Selective agar, such as Sabouraud dextrose (2%) agar supplemented with chloramphenicol and gentamicin (to allow the elimination of bacterial contaminants), fosters its growth. Colonies typically emanate a velvety texture, exhibiting hues ranging from green to brown. The length of incubation serves as a crucial determinant, with colonies emerging within five to seven days. Phenotypic identification relies on microscopic and macroscopic characteristics, encompassing features like pigmentation, distinctive conidial arrangement, and different degrees of sporulation or growth at different temperatures [77]. Microscopic research can be conducted via fluorescence (blancophore stain) and classic microscopy on lactophenol supplemented media. These features might help identify some cryptic species; nevertheless, they vary greatly according to different media conditions, often lacking phenotypic distinctions within a given *Aspergillus* section. Therefore, the phenotypic method should not be considered sufficient for cryptic species identification.

Serological tests, like the serum beta-1,3-D-glucan (BDG) and galactomannan (GM) enzyme immunoassay, may possess diagnostic value for invasive aspergillosis, with GM best performances on broncho-alveolar fluid rather than on serum [78]. However, sensitivity and specificity vary. Host factors, crucial in fungal infection predisposition, should always be integrated into the diagnostic algorithm to enhance the predictive value of serological tests [79].

Molecular methods’ advent brought the first revolution in fungal diagnostics [25]. Polymerase Chain Reaction (PCR) and DNA sequencing techniques target specific genetic markers such as: (i) fungal 18S or 23S ribosomal DNA (for broader at-section-level pan-fungal identification), (ii) Nuclear Ribosomal Internal Transcribed Spacer region (ITS, universally used as barcoding gene because of being a common DNA spacer in fungi) [38], (iii) beta-tubulin (benA, a component of eukaryotic cytoskeleton and present in multiple copies in the genome, with both conserved and variable regions) [80], (iv) calmodulin gene (cmdA, eukaryotic intracellular Ca2+ receptor, easily amplifiable with both conserved and variable regions) [32], (v) Mini-chromosome maintenance protein (mcm7, single copy gene encoding essential component for replication) [38]. Numerous other, more or less specific genes could target alternative metabolic pathways, such as toxin or pheromone production. However, to achieve differentiation within the section, it will still be necessary to integrate multiple targets (multiplex assays), resulting in a labor-intensive technique that is neither time-efficient, nor cost-effective, nor universally available [80].

MALDI-TOF has recently emerged as a powerful and relatively cost-effective tool, offering rapid and accurate species-level identification by analyzing protein profiles [81,82,83].

Another essential tool is the Mass Spectrometry Identification (MSI) platform, an independent and freely accessible online mass spectrometry database, which is helpful for all clinicians to correctly determine whether their isolates are cryptic *Aspergillus* species [84].

Even though advances have been made in this field and MALDI-TOF databases keep being updated, due to the high inter- and intra-variability of traits, a combination of methods may sometimes be recommended to achieve robust results and ensure the possible discovery of new species.

The correct identification has clinical relevance. Imbert et al., in a prospective multicenter study, analyzed 369 cryptic *Aspergillus* species, 15 responsible for invasive aspergillosis, showing a high rate of in vitro low susceptibility to antifungal drugs [85]. They also highlighted the correlation between pre-exposure to azoles and cryptic invasive aspergillosis [85].

## 6. Antimicrobial Susceptibility Testing

The landscape of AST involves several methodologies with sometimes intricate interpretations and often a lack of standardization [86]. Broth microdilution assays (e.g., Sensititre™ YeastOne™, Thermo Fisher Scientific (Waltham, MA, USA); Micronaut-AM, Bruker (Billerica, MA, USA)) contain the MICs, offer quantitative insights, and presently serve as the standard method of reference both for the European Committee on Antimicrobial Susceptibility Testing (EUCAST) [87] and by the Clinical Laboratory Standard Institute in the U.S. (CLSI) [88]. While E-test strips and gradient diffusion methods can visually illustrate susceptibility patterns, the interpretation remains challenging. Only occasionally has the E-test been shown to outperform broth microdilution. Furthermore, the lack of uniformity in testing and interpretation criteria makes them poor candidates for universal AST. Studies on flow cytometry mainly focused on *Candida* species, and showed promising results. Even though the lack of standardization and the significant error reported posed some concerns [86], similar studies on *Aspergillus* are lacking.

The intricate relationship between cryptic aspergillosis and antifungal susceptibility creates a complex interplay. While some groups (mostly *A. fumigatus* complex) can present a species-dependent susceptibility profile, in other groups (such as *A. niger* complex), strains from the same species show different patterns of resistance [24]. Another example is *A. lentulus*, which has demonstrated high MIC values for azoles and Amphotericin B (AMB), unlike *A. fumigatus* [89], and it is also reported to have poorer clinical outcomes [90].

## 7. Resistance Mechanisms

The clinical course of aspergillosis depends on the patient’s underlying condition, the identified *Aspergillus* species, the antifungal resistance profile, and treatment choice. Identifying the mold isolates to determine the antifungal susceptibility pattern and find the possible presence of intrinsic or acquired resistance is essential [41] to reduce the risk of treatment failure. Azoles that inhibit ergosterol synthesis and impede fungal growth, such as fluconazole (FCZ), itraconazole (ITC), voriconazole (VRC), isavuconazole (ISAV), and posaconazole (POS), are common antifungal drugs used for treatment in most cases [91,92]. Long-term use of azole drugs and extensive application of fungicides in agriculture represent the primary reasons for the emergence of azole resistance [60,93]. Additionally, some species have intrinsic resistance to antifungal-specific medicines: for example, *A. terreus* is intrinsically resistant to AMB [94], and *A. flavus* to polyenes [95]. Others are susceptible to a particular class of drug but may become resistant due to the prolonged incomplete dosages of antifungal [96]. Since the first azole-resistant *A. fumigatus* isolate was detected in 1997 the United States (U.S.), azole resistance has become increasingly reported in many countries, particularly in the Netherlands [97]. Azole-resistant isolates of *A. fumigatus* ranged from 6.6% to 28% worldwide [96].

**Figure 3:**
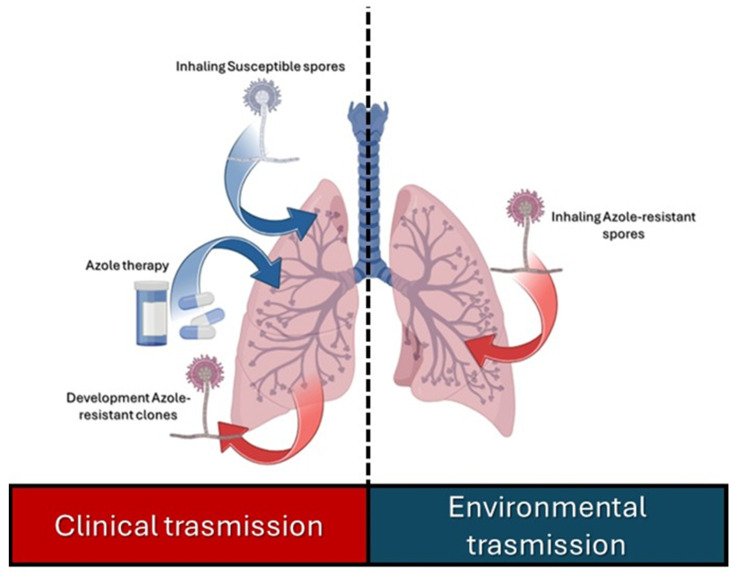
Clinical and environmental azole-resistant route of transmission.

Azoles are competitive inhibitors of the Cyp51A protein, a fundamental enzyme with lanosterol-14α-demethylase activity in the ergosterol biosynthesis of fungi [98]. Several mutations can affect azole activity and have been observed during prolonged antifungal therapy or prophylaxis [99,100]. The azole resistance has been related to a single allele of cyp51A, termed TR/L98H [98]. The allele contains a tandem repeat in the cyp51A promoter region combined with a single amino acid exchange of leucine 98 to histidine. The TR/L98H allele has been reported to occur worldwide in patients and the environment [101,102]. Clinical and environmental azole-resistant route of transmission is reported in Figure 3.

Azole resistance is a common finding, but the mutation of the Cyp51A gene has not yet been studied in all cryptic species [103]. *A. lentulus* has shown a mutation of this gene, which appears to differ from that highlighted for *A. fumigatus* [104]. *A. terreus* has also shown a mutation of Cyp51A gene, together with other resistant mechanisms, such as efflux pumps and hyperexpression of Cyp51A [72]. The development of azole resistance is most common in patients that have long-term azole history exposure, including patients with allergic and chronic pulmonary aspergillosis, individuals receiving prophylaxis, and those with predisposing conditions, such as preexisting lung cavities and cystic fibrosis [105]. The common use of azoles in agriculture and veterinary medicine affects the risk of inhaling conidia that already harbor azole resistance mechanisms [106].

FCZ and POS, often used in prophylaxis regimens among Hematopoietic stem-cell transplantation (HSCT) patients, are more likely to show higher MICs in cryptic species. Nevertheless, some strains might still show higher MICs for POS than ITC or VOR [24].

Escribano et al. observed that, in Spain, the prevalence of azole resistance in *A. fumigatus sensu lato* was 7.4% (63/847) [107]. However, azole resistance was higher among cryptic species (18/19, 95%) than in *A. fumigatus sensu stricto* (45/828, 5.5%). This higher prevalence of resistance was principally associated with the TR34/L98H mutation. Tsang et al. showed that most cryptic species identified were non-wild-type or resistant to azole. Notably, *A. tabacinus* and *A. tubingensis* were resistant to ISAV, VRC, POS and ITC. In addition, *A. austroafricanus* isolate fell into the area of technical uncertainty (ATU) for VRC. Moreover, *A. austroafricanus* and *A. sydowii* were resistant to ISAV [108].

Pinto et al. noted a 7.5% prevalence of cryptic species in the north of Portugal. *A. welwitschiae* (3.1%) and *A. lentulus* (2.2%) were the most frequent molds isolated. Most cryptic spies showed worrisome resistance to triazole (VRC 47.1%, POS 82.4%, and ISAV 100%) [29].

AMB is a broad-spectrum polyene binding to ergosterol and can cause cell membrane depolarization and damage membrane permeability, resulting in fungal cell death [109]. Some studies have also suggested that AMB can induce oxidative killing mechanisms in cell membranes and DNA by producing reactive oxygen species (ROS) [110,111].

Among *Aspergillus* spp., *A. terreus* is the strain harboring intrinsic resistance to AMB [112], as previously written. The mechanism related to AMB resistance in *A. terreus* and other *Aspergillus* species is not completely known. However, AMB resistance in the section *Terrei* seems to be associated with the modulation of chaperones, targeting ROS by mitochondria and influencing cellular redox homeostasis [113]. Other studies suggested that the resistance could be related to the glucan content in the cell wall or ergosterol content in cell membranes [114].

In Europe, AMB resistance is 2.6% to 10.8% for *A. fumigatus*. In France, *A. flavus* isolates show a worrisome rate of 84% of AMB-resistant strains. *A. niger* AMB-resistant isolates ranged from 75% to 12.8% in Greece and Belgium, respectively [113].

In different cryptic isolates, such as *A. lentulus*, *A. fumigatiaffinis*, *A. udagawae* (from *A. fumigatus* complex) and *A. alliaceus* (from *A. flavus* complex), AMB showed high MIC values [22,89,115,116]. In a recent article, Imbert et al. demonstrated a consistent reduction in susceptibility to AMB in Fumigati cryptic molds. However, Won et al. showed that AMB resistance is not so frequent in cryptic aspergillosis, except for *A. lentulus* [117].

## 8. Treatment

Cryptic *Aspergillus* species pose a significant treatment challenge, primarily due to their inherent resistance to widely used antifungal agents. Understanding the evolving landscape of antifungal therapy is critical in addressing the complexities introduced by these elusive fungal pathogens.

Historically, polyenes, like AMB, were fundamental in anti-mold therapy thanks to their broad-spectrum fungicidal action [118,119]. However, the significant toxicity and the logistical hurdles tied to intravenous administration have diminished their utility. These challenges necessitated the exploration of alternative therapeutic options. The advent of liposomal amphotericin B (LAMB) enhanced safety profiles, yet the issue of intravenous administration persisted [119]. Despite this, it remains vital for managing infections caused by *A. niger*, notorious for its resistance to triazoles [120].

Echinocandins, known for their favorable safety profiles, play a limited role against invasive mold diseases caused by cryptic *Aspergillus* species, with their efficacy in this context still under investigation due to limited validating studies [49,121,122]. Some research suggests the potential of echinocandins in combination with VRC or as monotherapy in rare azole resistance, particularly against cryptic species exhibiting high MICs to conventional treatments [123].

The azole class of antifungals has been extensively researched for its activity against *Aspergillus* species, with VRC being internationally recommended as the primary treatment for invasive aspergillosis [120,124]. Despite its effectiveness, VRC’s safety concerns have led to the search for safer alternatives. ISAV, demonstrated as non-inferior to VRC in the SECURE trial and known for its favorable safety profile, has emerged as the preferred first-line therapy in invasive aspergillosis [125,126].

Nevertheless, the rise of azole-resistant strains, including cryptic species like *A. udagawae* and *A. lentulus*, which show high MICs to all azoles, presents a formidable challenge [127,128,129]. Cryptic species often display intrinsic resistance to several antifungal classes, rendering some treatments less effective than against *A. fumigatus sensu stricto* [29]. For instance, *A. lentulus* and *A. udagawae* exhibit reduced susceptibility across multiple antifungal classes, including azoles and polyenes. This intrinsic resistance significantly hampers treatment options, necessitating alternative or combination therapies, potentially linked to increased toxicity and costs. Moreover, diagnostic challenges in accurately identifying cryptic *Aspergillus* species, often misidentified as *A. fumigatus sensu stricto*, can lead to inappropriate antifungal use, contributing to resistance development and spread [49]. Additionally, the lack of standardized susceptibility testing for cryptic species complicates resistance pattern assessments, further challenging effective antifungal therapy selection.

New antifungal agents with promising efficacy against *Aspergillus*-resistant strains have been developed in light of these challenges. Fosmanogepix (FMGX), an antifungal prodrug inhibiting the Gwt1 enzyme, which is crucial for glycosyl-phosphatidyl-inositol anchor biosynthesis, shows broad-spectrum activity against *Aspergillus* species, including azole-resistant strains [130,131]. Its unique action mechanism offers a novel therapeutic avenue for infections resistant to current treatments.

Ibrexafungerp (IBX) is the first of a new class of antifungals, the triterpenoids, inhibiting 1,3-β-D glucan synthase, essential for fungal cell wall synthesis [132]. Its efficacy against azole-resistant *Aspergillus* strains and oral bioavailability makes it a promising candidate for treating cryptic aspergillosis, offering easier administration and potential for outpatient treatment [133,134].

Rezafungin (RZF), a next-generation echinocandin with a prolonged half-life, allows for once-weekly dosing [135]. Its potent in vitro activity against various *Aspergillus* species, including cryptic species such as *A. calidoustus*, *A. lentulus*, *A. thermomutatus*, and *A. udagawae*, and convenient dosing regimen could significantly improve patient adherence [136]. Olorofim (OLO), belonging to the novel orotomides’ class, targets dihydroorotate dehydrogenase, inhibiting pyrimidine synthesis through a unique action mechanism [137]. Its potent activity against a broad spectrum of *Aspergillus* species, including resistant strains, underscores its development significance for managing difficult-to-treat Aspergillosis cases [138].

ATI-2307, a novel aromatic diamidine pentamidine-like compound, disrupts the mitochondrial respiratory chain complexes III and IV [139]. Although data remain sparse, early indications suggest promising activity against *Aspergillus* spp., contributing to the expanding arsenal against fungal pathogens [140].

Conversely, opelconazole (OPZ), a new synthetic azole designed for topical and nebulized administration, may not be effective against cryptic aspergillosis due to the prevalent resistance within this class. For instance, its in vitro efficacy against *A. flavus* and *A. niger* is notably weak. Similarly, encochleated AMB, which offers the advantage of oral administration, demonstrates limited activity against *A. terreus*, *A. flavus*, and *A. nidulans*, underscoring the need for ongoing research and development in the fight against resistant fungal infections.

## 9. Conclusions

*Aspergillus* spp. is a genus that encloses many species worldwide, many of which are addressed as “cryptic”. The role of these species in human diseases is enormously increasing, causing difficult-to-treat infections due to their resistance profile and the demanding effort needed to recognize them.

The increased use in the agricultural and biomedical fields of antifungals increasingly selects resistant *Aspergillus*. Cryptic species, in some cases, show high rates of resistant patterns that significantly increase mortality and morbidity, especially in immunocompromised patients.

In the ever-evolving landscape of microbiology, cryptic aspergillosis unfurls its complexities. The arsenal of culture characteristics, diagnostic methodologies, and antifungal susceptibility elucidates the intricate dance between the pathogen and the clinician. Unraveling the enigma of cryptic aspergillosis demands a harmonious symphony of clinical acumen, diagnostic precision, and therapeutic finesse, ensuring the pursuit of the best patient outcomes in the face of this elusive fungal foe.

From our point of view, we suggest reflecting on the possibility of a cryptic *Aspergillosis* species infection whenever there is a discrepancy between susceptible isolates of *Aspergillus* spp. and therapeutic failure despite correct treatment. Also, whenever divergence emerges, it is necessary to work with the microbiology laboratory so that the isolate can be further studied with specific techniques, like molecular ones.

Eventually, knowing the strong relationship between the environment and possible human exposure, we recommend performing an extensive anamnesis regarding the life habits of patients suspected of *Aspergillosis* infection, forecasting probable antifungal resistance.

In conclusion, addressing aspergillosis caused by cryptic *Aspergillus* species can be challenging; available antifungal drugs are not always practical. The emergence of novel medications represents a significant advance. These developments offer hope for more effective treatment strategies, improved diagnostic accuracy, and potential combination therapies.

## Figures and Tables

**Table 1:** Most frequently reported cryptic *Aspergillus* species.

*Aspergillus* Section	Cryptic Species
Fumigati	*A. lentulus*, *A. fumigatus*, *A. thermomutans*, *A. udagawae*, *A. viridinutans*, *A. fumigatiaffini*, *A. novofumigatus*, *A. felis*, *A. Clavutus*, *A. tsurutae*, *A. arcoverdensis*, *A. ellipticus*
Flavi	*A. tamarii*, *A. alliceus*, *Aspergillus nomius*
Terrei	*A. carneus*, *A. alabamensis*
Nigri	*A. tubingensis*, *A. awamori*, *A. welwitschiae*, *A. acidus*
Versicolores	*A. sydowii*, *A. creber*
Circumdati	*A. persii*, *A. westerdijkia*
Usti	*A. calidoustus*, *A. insuetus*, *A. keveii*, *A. granulosus*, *A. pseudodeflectus*, *A. puniceus*

## Data Availability

Not applicable.
